# Treatment Response of Cystic Echinococcosis to Benzimidazoles: A Systematic Review

**DOI:** 10.1371/journal.pntd.0000524

**Published:** 2009-09-29

**Authors:** Marija Stojkovic, Marcel Zwahlen, Antonella Teggi, Kamenna Vutova, Carmen M. Cretu, Roberto Virdone, Polyxeni Nicolaidou, Nazan Cobanoglu, Thomas Junghanss

**Affiliations:** 1 Section of Clinical Tropical Medicine, University Hospital Heidelberg, Heidelberg, Germany; 2 Institute of Social and Preventive Medicine, University of Bern, Bern, Switzerland; 3 Clinical Trials Unit Bern, Bern University Hospital, Bern, Switzerland; 4 Department of Infectious and Tropical Diseases, University of Rome “La Sapienza,” Rome, Italy; 5 Department of Infectious Diseases, Parasitology and Tropical Medicine, University of Medicine Sofia, Sofia, Bulgaria; 6 “C. Davila” University of Medicine and Pharmacology, Colentina Teaching Hospital, Bucharest, Romania; 7 Azienda Ospedaliera “V. Cervello,” Department of Gastroenterology, Palermo, Italy; 8 University Hospital “Attikon,” Department of Pediatrics, Haidari, Greece; 9 Department of Pediatrics, Hacettepe Children's Hospital, Ankara, Turkey; University of Oklahoma Health Sciences Center, United States of America

## Abstract

Over the past 30 years, benzimidazoles have increasingly been used to treat cystic echinococcosis (CE). The efficacy of benzimidazoles, however, remains unclear. We systematically searched MEDLINE, EMBASE, SIGLE, and CCTR to identify studies on benzimidazole treatment outcome. A large heterogeneity of methods in 23 reports precluded a meta-analysis of published results. Specialist centres were contacted to provide individual patient data. We conducted survival analyses for cyst response defined as inactive (CE4 or CE5 by the ultrasound-based World Health Organisation [WHO] classification scheme) or as disappeared. We collected data from 711 treated patients with 1,308 cysts from six centres (five countries). Analysis was restricted to 1,159 liver and peritoneal cysts. Overall, 1–2 y after initiation of benzimidazole treatment 50%–75% of active C1 cysts were classified as inactive/disappeared compared to 30%–55% of CE2 and CE3 cysts. Further in analyzing the rate of inactivation/disappearance with regard to cyst size, 50%–60% of cysts <6 cm responded to treatment after 1–2 y compared to 25%–50% of cysts >6 cm. However, 25% of cysts reverted to active status within 1.5 to 2 y after having initially responded and multiple relapses were observed; after the second and third treatment 60% of cysts relapsed within 2 y. We estimated that 2 y after treatment initiation 40% of cysts are still active or become active again. The overall efficacy of benzimidazoles has been overstated in the past. There is an urgent need for a pragmatic randomised controlled trial that compares standardized benzimidazole therapy on responsive cyst stages with the other treatment modalities.

## Author Summary

Cystic echinococcosis (CE) is a parasitic infection of worldwide occurrence transmitted to humans by dogs. After infection cysts develop, mainly in the liver and lung. Ultrasound-based staging of cysts into active, transitional, and inactive has opened new venues for treatment and follow-up. Currently four treatment modalities are in use: (1) surgery, (2) percutaneous sterilization techniques, (3) chemotherapy with benzimidazoles, and (4) watch and wait for inactive cysts. However, evidence is insufficient for these four modalities, and determining individual treatment options for patients remains controversial. Medical treatment with benzimidazoles started in the 1970s. Important questions remain unanswered, however, such as efficacy stratified by cyst type and the duration of treatment. We therefore initiated EchinoMEDREV, a collaborative effort to collect individual patient data from patients treated with benzimidazoles and to analyze cyst outcome after initiation of benzimidazole therapy using a common analytical strategy across treatment centres. We found that the efficacy of benzimidazoles has been overstated in the past. Additionally, natural cyst decay has not been taken into account. Evidence from randomized controlled trials is urgently needed to determine the true efficacy of benzimidazoles. Our analysis will help to design benzimidazole trial arms on the basis of the currently available best evidence.

## Introduction

Cystic echinococcosis (CE, hydatid disease) is a parasitic disease of worldwide prevalence. Hydatid cysts occur mainly in the liver (70%) and the lung (20%). Clinical symptoms and signs depend on their localisation, size, and number. Currently four treatment modalities are in use: (1) surgery, (2) PAIR (puncture, aspiration, injection of protoscolicidal agent, reaspiration), (3) chemotherapy with albendazole (ABZ) or mebendazole (MBZ), and (4) watch and wait for inactive, clinically silent cysts. The evidence supporting any of the four treatment modalities, from carefully designed clinical studies, is insufficient, and choosing treatment options for patients remains controversial [1].

The use of benzimidazoles in CE treatment started in the 1970s with MBZ. In the early 1980s ABZ was introduced and since then has largely replaced mebendazole. The main advantages of ABZ are claimed to be a lower dosage and better intestinal absorption. In treatment centres MBZ and ABZ are given at the World Health Organisation (WHO) recommended dosages of (MBZ, 40–50 mg/kg/day; ABZ, 10–15 mg/kg/day) [2]. Variability exists in the duration of treatment, which remains undefined. Duration of treatment is determined according to the ultrasound-based treatment response, resulting in repetitive treatment, which is part of our analysis.

Chemotherapy for the treatment of CE was initially recommended for inoperable patients and patients with multiple organ disease [2],[3]. Over the past decade several studies, mainly case series, have been published suggesting that chemotherapy could be an alternative to surgery in patients with uncomplicated cysts, leading to an increased use of chemotherapy over the years [4].

After more than 30 y of benzimidazole use, the following crucial question remains unanswered: what is the efficacy of benzimidazoles stratified by type and size of cysts, daily dose, and duration of treatment?

This project started with a systematic review of the published literature on the efficacy of treating CE with benzimidazoles. We had to conclude, however, that we could not obtain a clear picture of the long-term outcome of benzimidazole treatment because inclusion criteria, treatment, outcome measures, and follow-up of published studies varied widely with substantial overlap of cohorts [1], thus precluding a meta-analysis of published results. We therefore initiated EchinoMEDREV, a collaborative effort of CE specialists, to collect individual patient data from patients treated with benzimidazoles.

The main objectives of this collaborative study were to describe cyst outcome after initiation of benzimidazole treatment, with outcome defined by cyst stage determined by ultrasound following the WHO classification of 2001 [3], and to explore differences in outcome by cyst stage and size at initiation of treatment by using a common analytical strategy for all data across treatment centres.

## Methods

### Literature Search

A systematic search of MEDLINE, EMBASE, CCTR, and SIGLE was carried out from their inception until week 4 of 2004. The search was performed by a research librarian using the following search terms: echinococcosis, albendazole, mebendazole, hydatid disease, cystic echinococcosis. We also searched reference lists and asked researchers in the field for additional studies. No language restriction was used. Abstracts were screened for suitability by MS. The eligibility of studies was assessed independently by two investigators (TJ and MS). We included all types of study design with a minimum of 30 patients treated either with ABZ or MBZ. Studies in which drug treatment was an adjunct to surgery, PAIR, or a second drug were excluded.

### Individual Patient and Cyst Data Collection

The studies identified in the literature search revealed that there were large differences in baseline assessment of cyst stages, interventions (dose and duration of chemotherapy), length of follow-up, and outcome measures between published trials. These differences precluded the possibility to perform a meta-analysis of published results. Therefore we decided to collect individual patient data from the identified centres and initiated the EchinoMEDREV project.

Study centres that had conducted clinical studies on benzimidazole treatment of CE were contacted and asked to contribute published and unpublished individual patient data of benzimidazole-treated CE patients. Data extraction forms were developed, piloted, and revised. Data collection started in June 2005 and ended in December 2007. Data were extracted from patient charts at the individual treatment centres. Data collected were: demographic data (age, sex); treatment data (MBZ, ABZ, dosage, and duration of treatment, side effects, previous treatments); imaging data (initial ultrasound staging and staging at follow-up visits). The forms were sent to the coordinating centre at the University Hospital in Heidelberg where data were electronically entered into a database with EpiData, using data entry checks. Accuracy in data entry was checked by double entry verification. A final dataset was created after correcting detected data entry errors and exported to Stata for statistical analysis. Patients with single or multiple hydatid cysts were eligible. Cyst stage had to be recorded at the beginning and at least once after completion of the initial treatment episode. The minimum follow-up period was 1 y after completion of initial treatment. Cyst activity had to be assessed by ultrasonography and classified according to WHO (CL–CE5 or active [A]/transitional [T]/inactive [I]), Gharbi, Perdomo, or Caremani (Table 1).

**Table 1:** Ultrasound classification systems of CE cysts.

Gharbi et al. 1981 [32]	Perdomo et al. 1995 [33]	Caremani et al. 1997 [34]	WHO 2001	
G I, G III	P 1P 1a, 1b, 1cP 2	C IaC IbC IIa, IIb	CLCE1CE2	A, active: unilocular simple cyst with uniform anechoic content with or without visible cyst wall or multivesicular, multiseptated cyst, presence of daughter cysts, round or oval
G II, G IV	P 3	C IIIa, IIIb; C IV	CE3	T, transitional: unilocular cyst may contain daughter cysts, anechoic content detachment of membrane, form may be less rounded
G V	P 4, 4aP 5, 6	C Va, VbC VI, VIIa,VII b	CE4CE5	I, inactive: heterogenous, hypoechoic, or hyperechoic degenerative content, no daughter cysts, calcification of cyst

### Data Analysis

The analysis presented here includes only liver and peritoneal cysts (70%–75% of all CE cysts in humans), which were assessed by ultrasonography, and excluded lung cysts as they are not usually assessed by ultrasonography.

The cyst was used as the unit of analysis for a description of achieved outcomes, and the presence of multiple cysts was not taken into account. Data were analysed by intention-to-continue-treatment, ignoring treatment changes (MBZ/ABZ), interruptions, and subsequent treatment episodes.

We analysed several endpoints. First, initial treatment success for a cyst was defined as transformation from an initially active or transitional stage to an inactive stage or disappearance of the cyst (see Table 1 for classification based on ultrasonography). For this analysis time was measured from the start of first documented treatment to the date the stage was assessed as inactive or as disappeared or to the last documented assessment. Second, an analysis was made of the time for a cyst to become active again after the cysts had been staged as inactive; a necessary step, as some cysts that had reached an inactive stage had subsequently been staged as active again upon ultrasonography. For this analysis time was measured from the first (or second, or third) date at which a cyst was staged as inactive until the cyst was staged again as active. For these separate endpoints we performed time-to-event analyses using the Kaplan-Meier method and calculated the cumulative incidence of the events by subtracting the Kaplan-Meier survival estimate from one. Descriptive figures are presented stratified by centre where appropriate.

Despite the fact that all previous studies on CE cyst development had treated cysts as an independent unit of analysis even if multiple cysts were present in the same patient, we addressed clustering of cysts within patients. The question of heterogeneity by centre was also examined with data from several treatment centres. When addressing clearly specified hypotheses—such as the association of cyst size and time to inactivity or cyst disappearance—Cox proportional hazards models were fitted and a robust variance estimator was used [5] to account for the clustering of cysts within patients. In addition indicator variables were included for each centre. Two questions were investigated using robust Cox proportional hazards model:(1) the association of cyst CE stage at baseline with time to first inactivity or disappearance, and (2) the association of cyst size (<6 cm versus >6 cm) with time to inactivity or cyst disappearance. For the second question the first year (day 0 to day 365) and the follow-up time after year one (day 366 onwards) were analysed separately, because descriptive cumulative incidence plots hinted at the possibility that the cyst size mattered only after year one. Wald test-based *p*-values were calculated to obtain a hypothesis test for a whole group of indicator variables to be included in the robust Cox proportional hazards model. *p*<0.05 was considered statistically significant. For a general description of patients and cysts included in this analysis, counts and mean and standard deviation are provided where appropriate.

After observing and describing a multiphase phenomenon of cyst response to treatment, we additionally performed a simulation of the fate of 5,000 hypothetical cysts starting at treatment initiation and moving across the different phases of becoming inactive and relapsing to active. We made the simplifying assumption that phase durations can be described by an exponential distribution. We took the median and/or the 25th percentile obtained from the cumulative incidence estimates for the transitions from active to inactive and for the transitions from inactive to active. We then derived the lambda parameter of the exponential distribution via standard formulae: median, lambda = median/ln(2); 25th percentile, lambda = p25/ln(1/0.75). We further assumed that the duration of the next phase is independent of the duration of the previous phase. For each simulated cyst we assessed the current stage at year 1 to year 5 after treatment initiation. All analyses were performed using Stata Version 10 (StataCorp).

## Results

**Figure 1:**
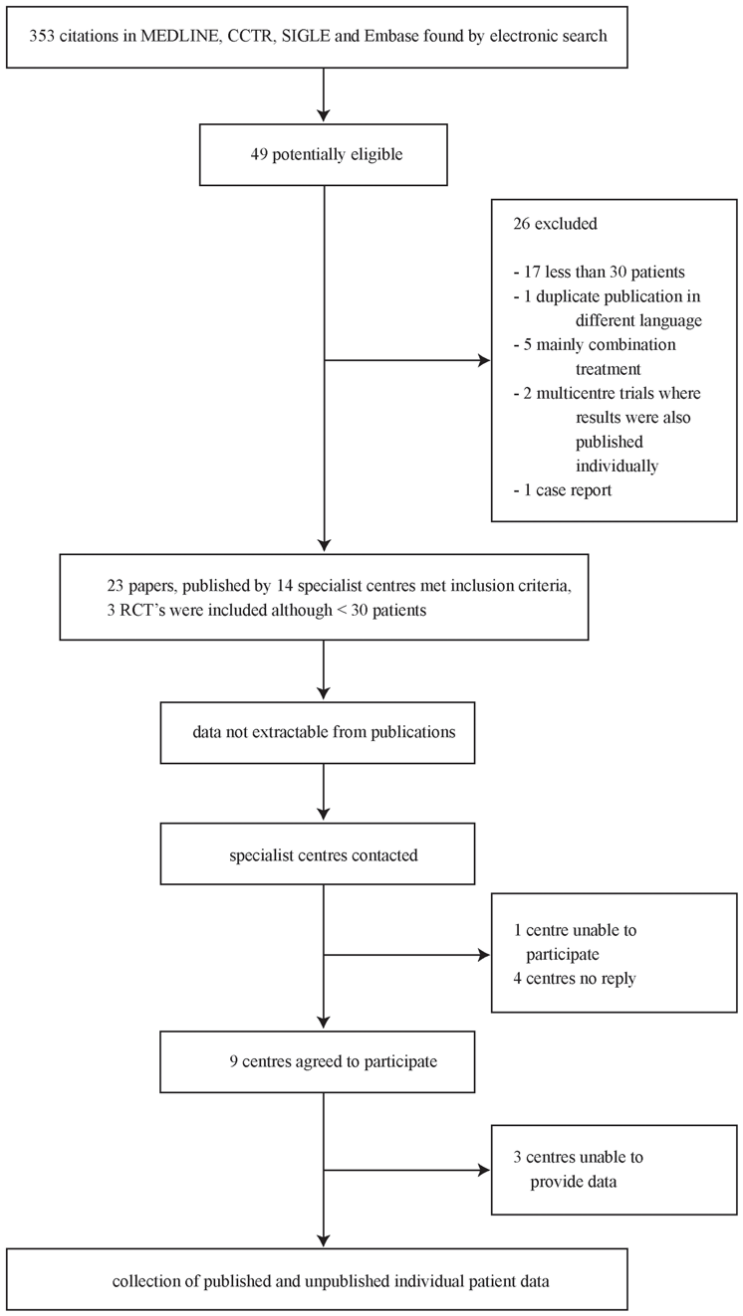
Flow diagram of searches, study selection, and specialist centre identification.

Out of 353 citations identified, 23 papers met the inclusion criteria (Figure 1). Three publications were randomised controlled trials [6]–[8], all other studies included were prospective or retrospective case series [9]–[28]. 19 publications were in English, one was written in Romanian [19], one in Chinese [23], one in Russian [25], and one in Italian [15]. Tables 2 and 3 summarize the characteristics of publications by the specialist centres identified through the literature search.

**Table 2:** Characteristics of study groups identified by the literature search where data were available.

Study Group Location	Data Available	Design	*n* Patients	Intervention ABZ/MBZ	Follow-up (mo)
Italy/Rome	Franchi et al. 1999 [14]	PCS	448	MBZ or ABZ	12–168
	De Rosa et al. 1996 (Italian, abstract only) [15]	Not clear from abstract	425	MBZ orABZ	Not clear from abstract
	Teggi et al. 1993 [16]	PCS	337	MBZ or ABZ	6–103
	Teggi et al. 1990 [17]	PCS	50	ABZ	6–42
Bulgaria	Vutova et al. 1999 [9]	PCS	53	MBZ	36–84
	Todorov et al. 1992 [10]	PCS	51	MBZ or ABZ	12–48
	Todorov et al. 1992 [11]	PCS	79	MBZ or ABZ	Mean 33
	Todorov et al. 1990 [12]	PCS	55	MBZ or ABZ	Mean 28
Romania	Radulescu et al. 1997 (Romanian) [19]	PCS	67	ABZ	24
Italy/Palermo	Sciarrino et al. 1991 [18]	CS	50	ABZ	Unclear
Greece	Messaritakis et al. 1991 [13]	PCS	39	MBZ	Mean 63±24
Turkey	Göcmen et al. 1993 [20]	PCS	56	MBZ	16–50

Abbreviations: CS, case series; PCS, prospective case series.

**Table 3:** Characteristics of study groups identified by the literature search where no data were available or no contact could be established.

Study Group Location	No Data Available/No Contact Established	Design	*n* Patients	Intervention ABZ/MBZ	Follow-up (mo)
Israel	Nahmias et al. 1994 [21]	CS	68	ABZ	36–84
China	Chai JJ et al. 2004 [24]	PCS	264	ABZ emulsion	24–48 y
	Chai JJ et al. 2003 [23]	CS	497	ABZ emulsion	Unclear
	Chai JJ et al. 2002 [22]	PCS	212	ABZ emulsion	12–24
	Wen H et al. 1994 [28]	PCS	58	ABZ	36–84
Russia	Shcherbakov AM et al. 1993 [25]	CS	53	MBZ	6–72
Libya	El-Mufti M et al. 1993 [26]	CS	63	ABZ	24
Great Britain	Horton RJ 1989 [27]	CS	253	ABZ	24–62
Spain	Gil-Grande LA et al. 1993 [6]	RCT	55	ABZ	12
Iran	Keshmiri M et al. 2001 [7]	RCT	17	ABZ	48
	Keshmiri M et al. 1999 [8]	RCT	20	ABZ	15–48

Abbreviations: CS, case series; PCS, prospective case series; RCT, randomised controlled trial.

**Figure 2:**
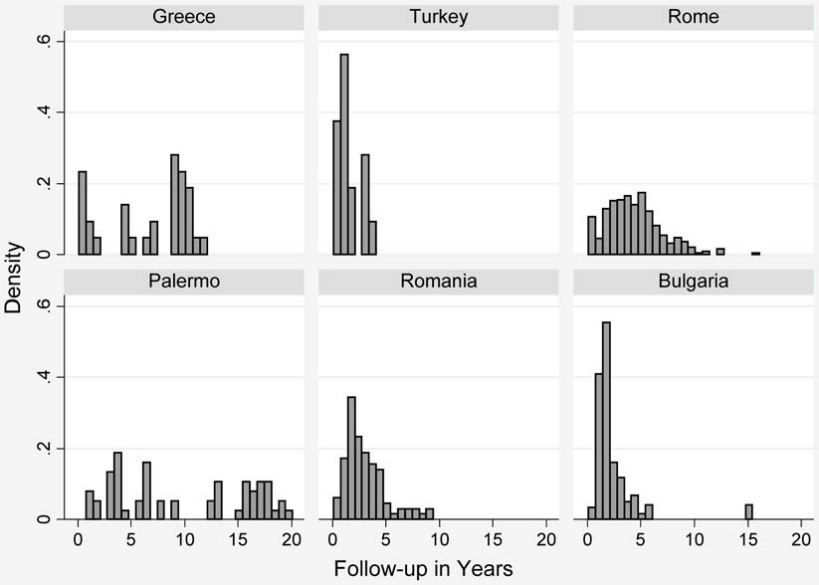
Length of follow-up per centre.

All 14 specialist centres identified were contacted: no reply was received from four centres [7], [8], [22]–[26]; one centre was unable to participate [6]; and nine centres agreed to participate. Of these nine centres six provided data [9]–[20], the others were unable to provide data [21],[27],[28]. In total we received data on 711 patients with 1,308 cysts. Table 4 summarizes the characteristics of patients with liver and peritoneal cysts. Table 5 shows cyst and follow-up details. Figure 2 shows length of follow-up per centre.

**Table 4:** Patient and treatment characteristics by centre: restricted to patients with liver and peritoneal cysts.

Centre	*n* Patients (Total 612)	Age (y) Mean±SD	Sex		Previous Treatment (Medical or Surgery)	*n* Treatment Courses	ABZ	MBZ	ABZ after MBZ	Drug Side Effects (Over All Treatment Episodes)							
			M	F						Raised Liver Enzymes		Low WBC		Low Platelets		Hair Loss	
										ABZ	MBZ	ABZ	MBZ	ABZ	MBZ	ABZ	MBZ
Rome	414	46.8±14.9	149	265	243	2.4±1.3	290	15	109	106	2	7	0	9	0	27	1
Bulgaria	73	42.2±18.1	33	40	39	1±0	51	22	—	4	0	0	0	0	0	5	0
Romania	59	40.4±16.6	30	29	31	1.11±0.37	59	—	—	5	—	1	—	0	—	0	—
Palermo	33	51.9±15.9	16	17	16	2.2±2.0	33	—	—	3	—	0	—	1	—	1	—
Greece	23	7.3±2.2	11	12	0	1.0±0.2	—	23	—	—	2	—	0	—	0	—	0
Turkey	10	7±3.3	3	7	2	1±0	7	3	—	0	0	0	0	0	0	0	0

Abbreviations: F, female; M, male; SD, standard deviation; WBC, white blood count.

**Table 5:** Cyst characteristics: restricted to patients with liver and peritoneal cysts and to patients where total days of treatment were available.

Centre	Total *n* Cysts (A+T) (1,159)	*n* Cysts per Patient Mean±SD	Size of Cysts Mean±SD (cm) First Visit	Cyst Stages at Presentation			Number of Follow-ups per Cyst	Follow-up Years per Cyst	Ultrasound Classification Used
				A	T	I^a^			
Rome	783	2.2±0.97	4.09±1.99	550	233	124	6.32±2.91	4.4±2.7	WHO
Bulgaria	176	2.7±2.03	3.97±2.05	175	1	3	3.73±1.46	2.3±2.5	WHO
Romania	96	1.63±0.74	5.11±3.81	90	6	0	6.40±2.08	3±1.9	WHO
Palermo	56	1.73±0.80	7.51±4.91	50	6	1	9.96±6.06	10±6.3	Gharbi
Greece	32	1.39±0.72	5.07±1.83	32	0	0	3.97±0.47	6.7±3.9	A/T/I
Turkey	16	1.6±0.84	4.48±2.04	15	1	0	4.81±1.38	1.5±1.2	A/T/I

a Inactive (I) cysts are cysts in patients with multiple cysts. The inactive cysts have not been the indication for treatment and have not been included in the analysis.

Abbreviation: SD, standard deviation.

The analysis presented here was restricted to patients with liver and peritoneal cysts, because of the reliability of ultrasound classification compared to other cyst locations. This restriction resulted in 1,159 cysts in 612 patients for analysis. Approximately 68% of data was obtained from one centre (Table 4).

**Figure 3:**
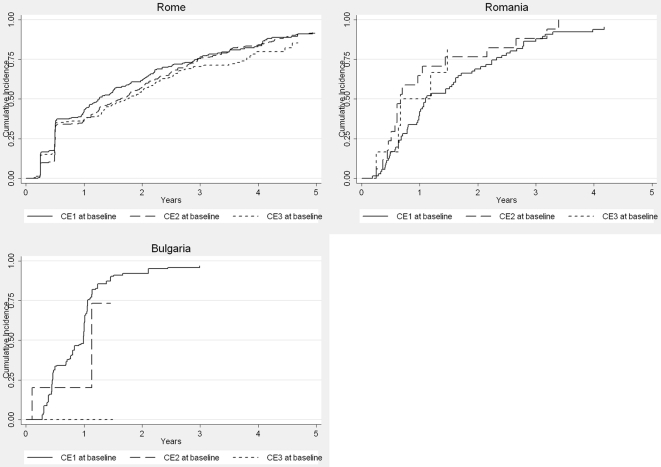
First time a cyst was staged as CE4, CE5, or disappeared by stage at baseline (only centres using the WHO CE classification).

Figure 3 shows the treatment response of individual cyst stages. Overall, 1–2 y after initiation of benzimidazole treatment 50%–75% of cysts initially staged as active in the CE1 category were staged as inactive or had disappeared compared to 30%–55% of CE2 and CE3 cysts. In the robust Cox proportional hazards model, CE3 stage cysts responded poorer than CE1 cysts from year one onwards (*p* = 0.043), but not up to year one (*p* = 0.43), and a centre effect was noted from year one onwards (*p* = 0.033, Wald test).

**Figure 4:**
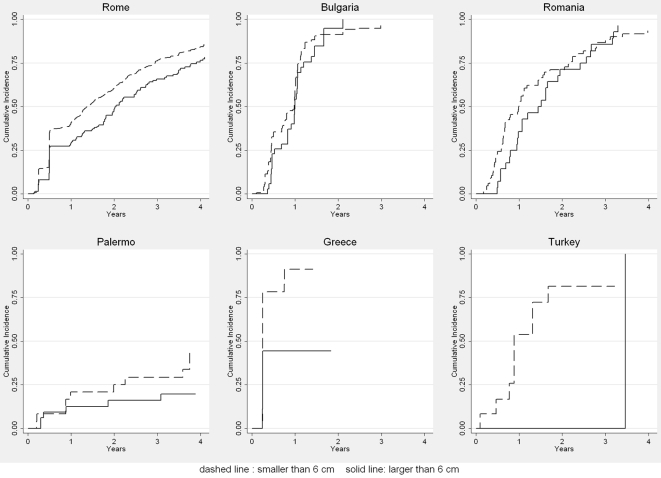
First time a cyst was staged as inactive or disappeared by size of cyst.

We further analysed the rate of inactivation/disappearance with regard to cyst size (Figure 4). Overall, 50%–60% of cysts <6 cm at baseline responded to treatment after 1–2 y, compared to 25%–50% of cysts >6 cm. In the robust Cox proportional hazards model cysts <6 cm responded better than larger cysts (*p* = 0.006) and a strong centre effect was noted (*p*<0.0001).

**Figure 5:**
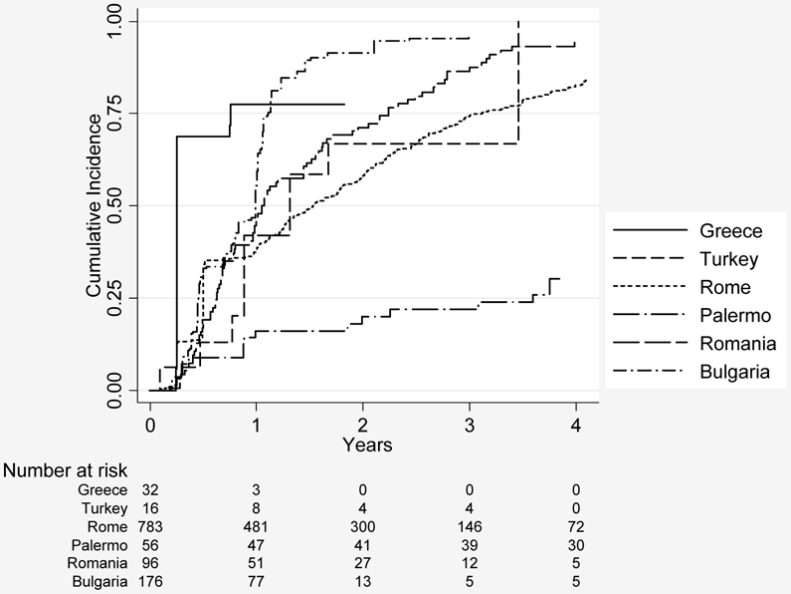
First time a cyst was staged as inactive or disappeared by centre.

Figure 5 shows the cumulative incidence of reaching an inactive stage or a disappearance of cysts for the first time by centres. Data from Greece and Bulgaria show inactivation/disappearance rates of cysts of 75%, increasing to around 90% within 2 y in Bulgaria. In contrast data from Palermo show inactivation or disappearance of cysts in approximately 20% of cases after 2 y. Data from Rome, Romania, and Turkey are between Greece and Palermo.

**Figure 6:**
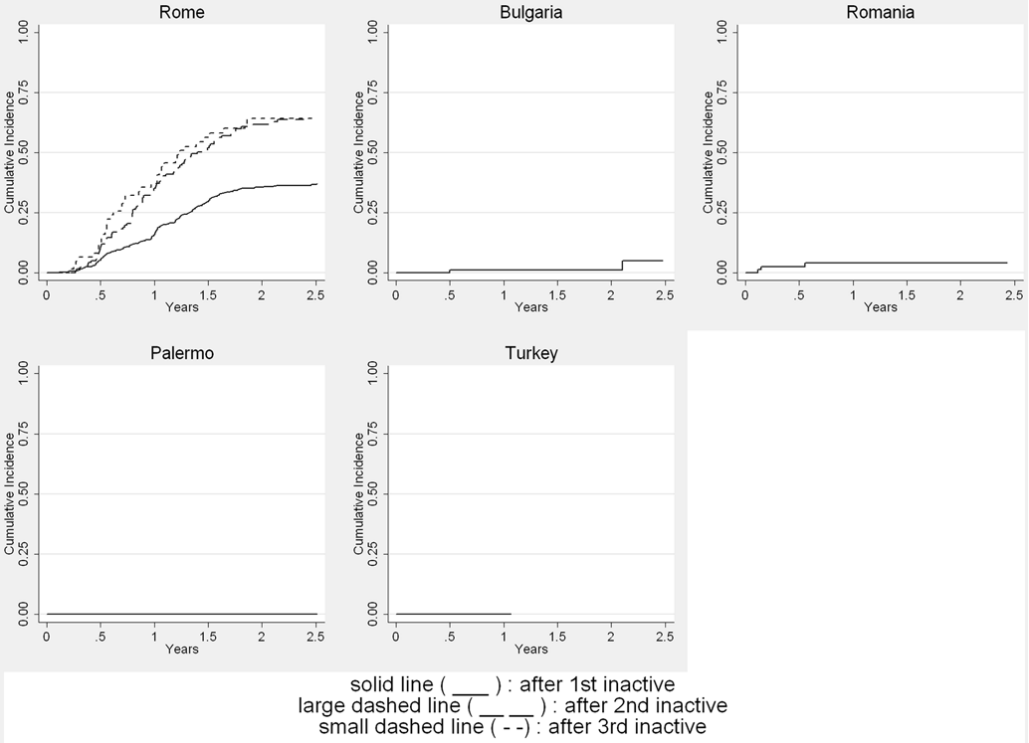
Time an active or transitional stage was reached stratified by the number of times an inactive stage had been reached previously (only centres that recorded recurrences are included).

Overall, cysts that reached an inactive stage for the first time relapsed (returned into an A or T stage) in around 25% of cases 2 y after inactivation (Figure 6). Cysts that reached an inactive stage for a second or third time showed relapse at a higher proportion and at an earlier stage: 60% of cysts relapsed within 2 y after the second or third inactivation.

**Figure 7:**
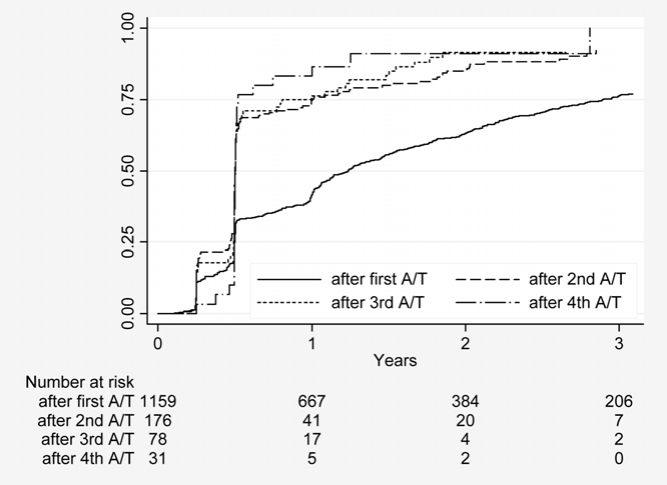
Time an inactive or disappeared stage was reached for the next time, stratified by the number of times an active or transitional stage had been reached previously.

Figure 7 shows the proportion of inactive/disappeared cysts over time stratified by the first, second, and third time the cysts started from A/T stages. The cumulative incidence curve after first A/I reflects what has been observed after treatment initiation. Cysts that were staged A/T for the second and third time were staged as inactive or as disappeared in about 75% and 85% 1 y later. These results were almost exclusively from the Rome centre.

In the simulation of hypothetical cysts, we estimated that 1 and 2 y after treatment initiation, 60% and 40% of cysts are still active or become active again.

## Discussion

In a collaborative effort, individual data from patients with CE were pooled from six centres in five countries and outcomes of liver and peritoneal cysts treated with benzimidazoles were described. We found a strong association between cyst activity and response to treatment, with a better response in highly active CE1 cysts, and an association in treatment response depending on the size of the cyst at the beginning of treatment, with cysts <6 cm in diameter responding better. Thus, our data suggest that small highly active cysts show the best initial treatment response. Overall 25% of cysts reverted to active status within 1.5 to 2 y after having initially responded, and multiple relapses were observed. We estimated that 2 y after treatment initiation 40% of cysts are still active or become active again. Our results are biologically plausible because early in the disease host response resulting in an increasing thickness of the pericyst and consolidation of cyst content has not yet reached a degree that prevents the drug to reach its target [29]. Additionally, it is important to note that natural decay is a component of the observed rate of inactivation. Available data suggest that this decay may be as high as 13.6% within 18 mo [29], and up to 20.6% within 44 mo [30]. This finding clearly leads to an overestimation of response to benzimidazole treatment as calculated from longitudinal data, which increase with the length of observation.

There are several limitations to this study. The published data collected from participating specialist centres are from case series. Results from case series are considered low level evidence in determining the efficacy of treatment options. In several analyses we found heterogeneity by centre. For example, the cumulative incidence curves for reaching an inactive cyst stage for the first time or the disappearance of a cyst after initial treatment showed large intercentre variability. Greek and Bulgarian data show a very rapid response, whereas data from Palermo show a very sluggish response to treatment. Time to first inactivation of cysts in the other centres looks quite similar. Rapid response to treatment in Greece and Bulgaria, however, remains unexplained. The particularly slow response to treatment shown in the dataset from Palermo could be due to the larger mean size of cysts at presentation, the difficulty of translating the Caremani classification into the WHO ultrasound classification (Table 1), and the well-known fact that inter-rater agreement between experts on classification of certain cyst stages is low, in particular for cysts containing daughter cysts—stage CE2 and stage CE3b according to WHO. Depending on the amount of consolidated matrix, cysts are either classified CE2 (daughter cysts with no matrix) or CE3b (daughter cysts with matrix).This distinction is important since the former is regarded active, the latter transitional [31]. Interobserver discrepancies occurred in the description of transitions from the inactive stages CE4 and CE5 to the active stages CE1 or CE2; some observers described these transitions and regard them as possible, whereas others do not. However, the number of misclassified cysts is not quantifiable in a retrospective dataset. It is very difficult, if not impossible, to consider all types and directions of biases when attempting to estimate the response of CE cysts to benzimidazoles from the available data. A very strong bias is certainly introduced by differing observation times with considerable impact on inactivation due to spontaneous involution. A final problem concerns data that we were unable to obtain (Table 2). Two centres that initially offered to deliver large datasets were eventually unable to do so. Two-thirds of our data have been provided by Rome, consequently the results are predominantly from one centre.

Despite these limitations, to our knowledge, this study represents the largest CE dataset ever collected and analyzed in a uniform approach; further it is likely the only dataset obtained from the main international specialist groups. The recommendations on benzimidazole treatment of CE are currently based on the published results from these centres. Through the collection of individual patient data and the pooled analysis of these data we have managed to overcome some of the existing limitations present in the published literature.

Does our study provide sufficient evidence to influence decisions for the treatment of CE? We think that our results are strong enough to cast doubts on overoptimistic expectations of the overall efficacy of benzimidazoles. When looking into substrata of the cyst population small CE1 cysts (diameter <6 cm) are a promising target for benzimidazole therapy, whereas stage CE2 and CE3 cysts respond poorly. The available evidence from this and other studies does not yet allow us, however, to formulate solid evidence-based drug treatment recommendations across all cyst stages, sizes, and locations. Our results highlight the urgent need to compare in a pragmatic randomised controlled trial the effect of standardized benzimidazole dose regimens on the individual active cyst stages (CE1, CE2, CE3a, and CE3b) substratified by cyst size. Such a trial would investigate as a primary outcome the proportion of cysts that become inactive (cyst stages CE4 and CE5) after treatment, and as a secondary outcome the yearly relapse rates up to 5 y after completion of treatment. The clarification of the efficacy of benzimidazoles in CE treatment is of paramount importance since benzimidazoles are the only drugs currently available to treat this neglected disease. Surgery as an alternative to benzimidazoles carries a significantly higher risk of adverse events, in particular intra- and postoperative morbidity and mortality and disseminated disease due to intraoperative spillage of viable hydatid material. Percutaneous fine needle techniques such as PAIR are only applicable to cyst stages CE1 and possibly CE3a, but not to CE2 and CE3b, which makes it necessary to explore large bore catheter techniques if albendazole turns out to be less effective in these cyst stages as suggested by our analysis.

## Supporting Information

Checklist S1 QUOROM checklist.
(0.17 MB PDF)
