# Supplementary material for: Treatment Response of Cystic Echinococcosis to Benzimidazoles: A Systematic Review
Source: PLoS Negl Trop Dis. 2009 Sep 29;3(9):e524. doi: 10.1371/journal.pntd.0000524 (PMC2745697; doi:10.1371/journal.pntd.0000524)
Supplement: Checklist S1 — QUOROM checklist. (0.17 MB PDF) [file pntd.0000524.s001.pdf]

Improving the quality of reports of meta-analyses of randomised controlled trials: the QUOROM statement checklist

Heading	Subheading	Descriptor	Reported? (Y/N)	Page number
Title		Identify the report as a meta-analysis [or systematic review] of RCTs[26]		
Abstract		Use a structured format[27]		
		**Describe**		
	Objectives	The clinical question explicitly	Y	2
	Data sources	The databases (ie, list) and other information sources	Y	2
	Review methods	The selection criteria (ie, population, intervention, outcome, and study design); methods for validity assessment, data abstraction, and study characteristics, and quantitative data synthesis in sufficient detail to permit replication	Y	2
	Results	Characteristics of the RCTs included and excluded; qualitative and quantitative findings (ie, point estimates and confidence intervals); and subgroup analyses	Y	2
	Conclusion	The main results	Y	2
		**Describe**		
Introduction		The explicit clinical problem, biological rationale for the intervention, and rationale for review		
Methods	Searching	The information sources, in detail[28] (eg, databases, registers, personal files, expert informants, agencies, hand-searching), and any restrictions (years considered, publication status,[29] language of publication[30,31])	Y	5
	Selection	The inclusion and exclusion criteria (defining population, intervention, principal outcomes, and study design[32]	Y	5
	Validity assessment	The criteria and process used (eg, masked conditions, quality assessment, and their findings[33-36])	Y	5
	Data abstraction	The process or processes used (eg, completed independently, in duplicate)[35,36]	Y	5,6
	Study characteristics	The type of study design, participants' characteristics, details of intervention, outcome definitions, &c,[37] and how clinical heterogeneity was assessed	Y	5,6 Table 3,4
	Quantitative data synthesis	The principal measures of effect (eg, relative risk), method of combining results (statistical testing and confidence intervals), handling of missing data; how statistical heterogeneity was assessed;[38] a rationale for any a-priori sensitivity and subgroup analyses; and any assessment of publication bias[39]	Y	7,8
Results	Trial flow	Provide a meta-analysis profile summarising trial flow (see figure)	Y	Figure 1
	Study characteristics	Present descriptive data for each trial (eg, age, sample size, intervention, dose, duration, follow-up period)	Y	23,24 Table 4,5
	Quantitative data synthesis	Report agreement on the selection and validity assessment; present simple summary results (for each treatment group in each trial, for each primary outcome); present data needed to calculate effect sizes and confidence intervals in intention-to-treat analyses (eg 2×2 tables of counts, means and SDs, proportions)	Y	9,10
Discussion		Summarise key findings; discuss clinical inferences based on internal and external validity; interpret the results in light of the totality of available evidence; describe potential biases in the review process (eg, publication bias); and suggest a future research agenda	Y	11, 12, 13

Quality of reporting of meta-analyses
